# Delays to Antibiotics in the Emergency Department and Risk of Mortality in Children With Sepsis

**DOI:** 10.1001/jamanetworkopen.2024.13955

**Published:** 2024-06-05

**Authors:** Roni D. Lane, Troy Richardson, Halden F. Scott, Raina M. Paul, Fran Balamuth, Matthew A. Eisenberg, Ruth Riggs, W. Charles Huskins, Christopher M. Horvat, Grant E. Keeney, Leslie A. Hueschen, Justin M. Lockwood, Vishal Gunnala, Bryan P. McKee, Nikhil Patankar, Venessa Lynn Pinto, Amanda M. Sebring, Matthew P. Sharron, Jennifer Treseler, Jennifer J. Wilkes, Jennifer K. Workman

**Affiliations:** 1 Division of Pediatric Emergency Medicine, Department of Pediatrics, Primary Children’s Hospital, University of Utah, Salt Lake City; 2 Children’s Hospital Association, Lenexa, Kansas; 3 Section of Emergency Medicine, Department of Pediatrics, University of Colorado School of Medicine, Aurora; 4 Pediatric Emergency Medicine, Children’s Hospital of Orange County, Orange, California; 5 Division of Emergency Medicine, Department of Pediatrics, Children’s Hospital of Philadelphia, University of Pennsylvania Perelman School of Medicine, Philadelphia; 6 Division of Emergency Medicine, Department of Pediatrics, Boston Children’s Hospital, Harvard Medical School, Boston, Massachusetts; 7 Department of Emergency Medicine, Boston Children’s Hospital, Harvard Medical School, Boston, Massachusetts; 8 Division of Pediatric Infectious Diseases, Department of Pediatric and Adolescent Medicine, Mayo Clinic College of Medicine and Science, Rochester, Minnesota; 9 Department of Critical Care Medicine, UPMC, Children’s Hospital of Pittsburgh, Pittsburgh, Pennsylvania; 10 Department of Pediatric Emergency Medicine, Mary Bridge Children’s Hospital, Tacoma, Washington; 11 Division of Emergency Medicine, Department of Pediatrics, Children’s Mercy Hospital, University of Missouri-Kansas City, Kansas City; 12 Section of Hospital Medicine, Department of Pediatrics, University of Colorado School of Medicine, Aurora; 13 Division of Critical Care Medicine, Phoenix Children’s Hospital, Phoenix, Arizona; 14 Division of Critical Care Medicine, Department of Pediatrics, Akron Children’s Hospital, Akron, Ohio; 15 Pediatric Critical Care, Baptist St Anthony’s Health System, Amarillo, Texas; 16 Division of Pediatric Critical Care, Department of Pediatrics, Baylor College of Medicine, Houston, Texas; 17 Division of Pediatric Critical Care, Department of Pediatrics, Atrium Health Levine Children’s, Charlotte, North Carolina; 18 Division of Critical Care Medicine, Department of Pediatrics, Children’s National Hospital, George Washington University School of Medicine, Washington, DC; 19 Program for Patient Safety and Quality, Boston Children’s Hospital, Boston, Massachusetts; 20 Division of Cancer and Blood Disorders, Department of Pediatrics, University of Washington School of Medicine, Seattle; 21 Division of Pediatric Critical Care, Department of Pediatrics, University of Utah, Salt Lake City

## Abstract

**Importance:**

Pediatric consensus guidelines recommend antibiotic administration within 1 hour for septic shock and within 3 hours for sepsis without shock. Limited studies exist identifying a specific time past which delays in antibiotic administration are associated with worse outcomes.

**Objective:**

To determine a time point for antibiotic administration that is associated with increased risk of mortality among pediatric patients with sepsis.

**Design, Setting, and Participants:**

This retrospective cohort study used data from 51 US children’s hospitals in the Improving Pediatric Sepsis Outcomes collaborative. Participants included patients aged 29 days to less than 18 years with sepsis recognized within 1 hour of emergency department arrival, from January 1, 2017, through December 31, 2021. Piecewise regression was used to identify the inflection point for sepsis-attributable 3-day mortality, and logistic regression was used to evaluate odds of sepsis-attributable mortality after adjustment for potential confounders. Data analysis was performed from March 2022 to February 2024.

**Exposure:**

The number of minutes from emergency department arrival to antibiotic administration.

**Main Outcomes and Measures:**

The primary outcome was sepsis-attributable 3-day mortality. Sepsis-attributable 30-day mortality was a secondary outcome.

**Results:**

A total of 19 515 cases (median [IQR] age, 6 [2-12] years) were included. The median (IQR) time to antibiotic administration was 69 (47-116) minutes. The estimated time to antibiotic administration at which 3-day sepsis-attributable mortality increased was 330 minutes. Patients who received an antibiotic in less than 330 minutes (19 164 patients) had sepsis-attributable 3-day mortality of 0.5% (93 patients) and 30-day mortality of 0.9% (163 patients). Patients who received antibiotics at 330 minutes or later (351 patients) had 3-day sepsis-attributable mortality of 1.2% (4 patients), 30-day mortality of 2.0% (7 patients), and increased adjusted odds of mortality at both 3 days (odds ratio, 3.44; 95% CI, 1.20-9.93; *P* = .02) and 30 days (odds ratio, 3.63; 95% CI, 1.59-8.30; *P* = .002) compared with those who received antibiotics within 330 minutes.

**Conclusions and Relevance:**

In this cohort of pediatric patients with sepsis, 3-day and 30-day sepsis-attributable mortality increased with delays in antibiotic administration 330 minutes or longer from emergency department arrival. These findings are consistent with the literature demonstrating increased pediatric sepsis mortality associated with antibiotic administration delay. To guide the balance of appropriate resource allocation with time for adequate diagnostic evaluation, further research is needed into whether there are subpopulations, such as those with shock or bacteremia, that may benefit from earlier antibiotics.

## Key Points

**Question:**

Is the timing of antibiotic administration associated with sepsis-attributable mortality in pediatric sepsis?

**Findings:**

In this multicenter cohort study of 19 515 pediatric patients with sepsis recognized within 1 hour of emergency department arrival, antibiotic administration beyond 330 minutes was associated with an increase in 3-day and 30-day sepsis-attributable mortality.

**Meaning:**

These findings suggest that long delays in antibiotic therapy are associated with increased risk of mortality among children with sepsis.

## Teaser

This cohort study examines the association of the timing of antibiotic administration in the emergency department (ED) with the risk of sepsis-attributable mortality in children.

## Introduction

Sepsis is the leading cause of death in children worldwide,^1^ with an estimated 3.4 million deaths in 2017.^2^ It is a major cause of pediatric morbidity^3^ and a substantial contributor to health care utilization^3,4,5,6,7^ and cost burden.^8,9^ Although not all sepsis is caused by bacteria,^10,11,12^ early antibiotic administration has been a longstanding, cornerstone recommendation for the treatment of sepsis by the Surviving Sepsis Campaign,^13,14,15^ the American College of Critical Care Medicine,^16,17,18^ and the American Heart Association Pediatric Advanced Life Support guidelines.^19^ The Pediatric Surviving Sepsis Campaign International Guidelines for the Management of Septic Shock and Sepsis-Associated Organ Dysfunction recommend antibiotic administration within 1 hour of recognition of septic shock and within 3 hours of recognition of sepsis-associated organ dysfunction without shock.^20^

Although the importance of timely antibiotics in pediatric sepsis is not in question, a precise understanding of timeliness has not been established in children. Prior studies^11,21,22^ evaluating the association of time to antibiotic administration with pediatric sepsis outcomes have been single-center investigations that may not have been powered to detect clinical differences at multiple time points. The objective of this study was to examine data from a cohort of children treated for sepsis as a part of a large, multicenter improvement collaborative to evaluate the association of the time from sepsis identification to antibiotic administration with sepsis-attributable mortality. Our first aim was to use time to antibiotic administration data to determine whether there was a time point past which antibiotic administration was associated with a change in sepsis-attributable mortality rates; if such a point existed, our second aim was to describe the association of time to antibiotic administration with sepsis-attributable mortality in children.

## Methods

### Design and Data Source

We conducted a multicenter, retrospective cohort study using data from the Children’s Hospital Association’s Improving Pediatric Sepsis Outcomes (IPSO) quality improvement collaborative,^23^ following the Strengthening the Reporting of Observational Studies in Epidemiology (STROBE) reporting guidelines for cohort studies.^24^ The Colorado Multiple Institutional Review Board found this study exempt from review because it is using secondary data for analysis, thus waiving the requirement for informed consent, in accordance with 45 CFR §46. The collaborative began in 2016 and was aimed at decreasing pediatric sepsis mortality in children’s hospitals and reducing hospital-onset sepsis through bundled care that emphasizes standardized timeliness for recognition and management. At the time of this analysis, there were 57 participating hospitals, ranging from large, academic, quaternary, freestanding children’s hospitals to smaller children’s hospitals within a hospital, located in both urban and suburban settings in the US.

The IPSO collaborative maintains a database of patients treated for sepsis based on data contributed electronically from participating sites. Patients were included in the IPSO database if they met previously published IPSO sepsis definitions, which are based on sepsis-specific recognition tools and intention-to-treat sepsis interventions (eAppendix in Supplement 1).^25,26^ IPSO critical patients with sepsis are a subset of the IPSO sepsis cohort (eAppendix in Supplement 1).^25^ The IPSO database includes an estimate of the time of sepsis recognition, known as functional time zero (FTZ), which is the earliest time of a sepsis screen, huddle, order set use, first intravenous (IV) fluid, or antibiotic administration (eAppendix in Supplement 1).^26^ For this study, we abstracted data from the IPSO database, including demographic, clinical, processes of care, and outcome variables. Patient sex was not a data element collected in the IPSO database.

### Patients

We included patients in the IPSO database aged 29 days to less than 18 years who presented to the emergency department (ED) with suspected sepsis from January 1, 2017, through December 31, 2021. From the full IPSO database, we restricted the study cohort to focus on a more uniform population of patients who had sepsis at ED presentation that was recognized quickly, and to minimize the impact of delayed sepsis recognition on outcomes. Thus, the cohort includes those with FTZ within 1 hour of ED arrival, where FTZ is based on a proxy for sepsis recognition (sepsis screen, huddle, or order set). Patients were excluded if their FTZ was based on interventions (first IV fluid bolus or antibiotic administration) or if they had antibiotics administered more than 7 hours after ED arrival. Initial data validation via medical record review (performed by R.D.L. and H.F.S.) at individual sites identified that antibiotics given at more than 7 hours did not usually represent the first antibiotic administration, but rather a subsequent dose after a first dose was given before arrival, or a perioperative or other prophylactic medication. Candidates were excluded if they were from centers with incomplete data, were transferred from an outside facility, had FTZ outside the ED, or lacked antibiotic administration time documentation (eFigure 1 in Supplement 1).

### Exposures and Outcomes

The primary exposure was the number of minutes from ED arrival to antibiotic administration (intravenous, intramuscular, or intraosseous). Other antimicrobial agents (eg, antifungal, antiparasitic, or antiviral) are not captured in the IPSO database. The main outcome was sepsis-attributable 3-day mortality. To determine sepsis-attributable mortality, each site developed internal standardized procedures for medical review of all mortalities to determine whether the death was related to the index sepsis event (eAppendix in Supplement 1).^23^ Patients experiencing death from other causes were not included in the mortality analysis. Our secondary outcome was sepsis-attributable 30-day mortality. Exploratory outcomes included intensive care unit (ICU) admission, vasoactive medication administration, ventilator use, placement of a central venous line during the hospital stay, length of stay (capped at 30 days), and ICU-free, ventilator-free, and vasoactive-free days (of 30 days).

### Statistical Analysis

Data analysis was performed from March 2022 to February 2024. We used nonlinear piecewise logistic regression to estimate an inflection point in antibiotic administration time (measured in 30-minute increments) where the linear association of time to antibiotic administration with sepsis-attributable 3-day mortality significantly changed. This approach is an iterative process that requires initial inputs for model intercept, preinflection slope, postinfection slope, and inflection point to derive model-based estimates and 95% CIs based on nonlinear least squares estimation. The initial input for inflection point was estimated through visual inspection of time series data. We estimated initial slope and intercept inputs before and after the initial inflection point input by running a logistic regression model with 3-day mortality as the primary outcome and time to antibiotic administration (measured as the number of 30-minute intervals from arrival) as a continuous covariate in the model. Then, using these initial parameter estimates, we derived a final estimate of the inflection point from the nonlinear model. We then tested for a significant change in slope before vs after the model-based inflection point using an interrupted time series approach. Because time increments with 0% mortality are ignored in this modeling approach, we replaced 0 values with mortality estimates of 0.001% to ensure inclusion in our model.

Demographic, hospital, and outcome data were summarized using absolute values (number) and proportions (percentages) for categorical variables, and medians with IQRs for continuous variables. We compared characteristics and unadjusted outcomes between IPSO cases with antibiotic administration before vs after the inflection point using Pearson χ^2^ tests for categorical variables and Wilcoxon rank-sum tests for continuous, nonnormal variables. Adjusted odds ratios (ORs) with 95% CIs were calculated for 3-day and 30-day sepsis-attributable mortality using multivariable generalized linear mixed modeling, assuming a binomial distribution and underlying logit link function, and included a random center effect to account for the clustering of sepsis episodes at the same center. In addition to time-to-antibiotic groups, initial model covariates (selected a priori) included age, high-risk conditions (malignant neoplasm, asplenia, bone marrow transplant, indwelling central line, solid-organ transplant, severe intellectual disability, immunocompromised status, and technology dependence), long-term ventilator dependence, bacteremia, initial lactate value, IPSO sepsis vs critical sepsis, time to IV fluid bolus, and FTZ source (screen, huddle, or order set) (eAppendix in Supplement 1). Subsequently, we tested hospital type, arrival time of day, and study year in the models (eAppendix in Supplement 1). Hospital type and time to IV fluid bolus were excluded because of multicollinearity. We used backward selection to remove any covariate with *P* > .20 to achieve model parsimony.

Post hoc subgroup analyses were performed among patients with bacteremia, patients with IPSO critical sepsis, and those with high-risk conditions (including those undergoing long-term ventilation) to investigate the inflection point in the association of antibiotic administration time with sepsis-attributable 3-day mortality. The piecewise regression analysis for patients with bacteremia was truncated at 180 minutes after ED arrival because there were no deaths in this subgroup after this time. In addition, we compared patients receiving antibiotics within the first 29 minutes with those receiving antibiotics at 30 to 329 minutes.

Analyses were completed using SAS statistical software version 9.4 (SAS Institute). Statistical significance was set at 2-sided *P *<* *.05.

## Results

There were 70 296 cases from 57 hospitals in the IPSO database at the time of data analysis. From these, 19 515 cases (median [IQR] age, 6 [2-12] years) from 51 hospitals were included in the study (eFigure 1 in Supplement 1), with the largest exclusions based on age (12 857 patients), non-ED FTZ (14 541 patients), and FTZ 1 hour or longer from ED arrival (11 529 patients). A total of 11 121 patients (57.0%) had at least 1 high-risk condition (Table 1). The median (IQR) time to antibiotic administration was 69 (47-116) minutes.

**Table 1.** Baseline Characteristics of Study Population

Characteristic	Patients, No. (%)		
	Total (N = 19 515)	Antibiotic <330 min (n = 19 164)	Antibiotic ≥330 min (n = 351)
Time to first antibiotic, median (IQR), min	69 (47-116)	68 (46-111)	364 (347-387)
Age at FTZ, median (IQR), y	6 (2-12)	6 (2-12)	7 (2-13)
Age group			
29-60 d	550 (2.8)	536 (2.8)	14 (4.0)
61-364 d	1603 (8.2)	1570 (8.2)	33 (9.4)
1-4 y	6170 (31.6)	6066 (31.7)	104 (29.6)
5-10 y	5052 (25.9)	4974 (26.0)	78 (22.2)
11-17 y	6140 (31.5)	6018 (31.4)	122 (34.8)
High-risk conditions			
Not reported	8394 (43.0)	8186 (42.7)	208 (59.3)
Malignant neoplasm	3660 (18.8)	3638 (19.0)	22 (6.3)
Asplenia	613 (3.1)	612 (3.2)	1 (0.3)
Bone marrow transplant	615 (3.2)	610 (3.2)	5 (1.4)
Indwelling line	4048 (20.7)	4021 (21.0)	27 (7.7)
Solid-organ transplant	617 (3.2)	611 (3.2)	6 (1.7)
Intellectual disability	3541 (18.1)	3479 (18.2)	62 (17.7)
Immunocompromised	4472 (22.9)	4440 (23.2)	32 (9.1)
Technology dependent	5130 (26.3)	5046 (26.3)	84 (23.9)
Long-term ventilation			
No	15 292 (90.5)	15 006 (90.5)	286 (93.5)
Yes	1604 (9.5)	1584 (9.5)	20 (6.5)
Not reported	2619 (13.4)	2574 (13.4)	45 (12.8)
Bacteremia			
No	15 874 (87.7)	15 591 (87.6)	283 (90.4)
Yes	2230 (11.4)	2200 (12.4)	30 (9.6)
Not reported	1411 (7.2)	1373 (7.2)	38 (10.8)
Lactate value, median (IQR), mg/dL	18 (9-27)	18 (9-27)	18 (9-27)
Lactate category			
≤36 mg/dL	11 087 (56.8)	10 970 (57.2)	117 (33.3)
>36 mg/dL	1371 (7.0)	1353 (7.1)	18 (5.1)
Not reported	7057 (36.2)	6841 (35.7)	216 (61.5)
Time to first hypotension (from arrival), median (IQR), min	68 (15-285)	68 (14-282)	101 (17-450)
Bolus volume, median (IQR), mL/kg			
Within 50 min	15 (0-20)	16 (0-20)	0 (0-0)
Within 170 min	40 (30-50)	40 (30-51)	38 (18-40)
Time to first bolus (>5 mL/kg), median (IQR), min	45 (31-69)	45 (30-68)	93 (56-156)
Time to FTZ from arrival, median (IQR), min	13 (8-23)	13 (8-23)	16 (11-28)
IPSO sepsis population			
IPSO sepsis	12 676 (65.0)	12 412 (64.8)	264 (75.2)
IPSO critical population	6839 (35.0)	6752 (35.2)	87 (24.8)
FTZ source			
Screen	14 293 (73.2)	14 000 (73.1)	293 (83.5)
Huddle	999 (5.1)	985 (5.1)	14 (4.0)
Order set	4223 (21.6)	4179 (21.8)	44 (12.5)
Year			
2017	2904 (14.9)	2850 (14.9)	54 (15.4)
2018	4366 (22.4)	4294 (22.4)	72 (20.5)
2019	4784 (24.5)	4700 (24.5)	84 (23.9)
2020	4126 (21.1)	4059 (21.2)	67 (19.1)
2021	3335 (17.1)	3261 (17.0)	74 (21.1)
Arrival time of day			
12:00 am-5:59 am	2400 (12.3)	2359 (12.3)	41 (11.7)
6:00 am-11:59 am	4309 (22.1)	4233 (22.1)	76 (21.7)
12:00 pm-5:59 pm	6671 (34.2)	6522 (34.0)	149 (42.5)
6:00 pmd-11:59 pm	6135 (31.4)	6050 (31.6)	85 (24.2)
Hospital type			
Freestanding children’s hospital (n = 25)	16 470 (84.4)	16 190 (84.5)	280 (79.8)
Not freestanding (n = 26)	3045 (15.6)	2974 (15.5)	71 (20.2)

Abbreviations: FTZ, functional time zero; IPSO, Improving Pediatric Sepsis Outcomes.

SI conversion factor: To convert lactate to millimoles per liter, multiply by 0.111.

**Figure 1.**
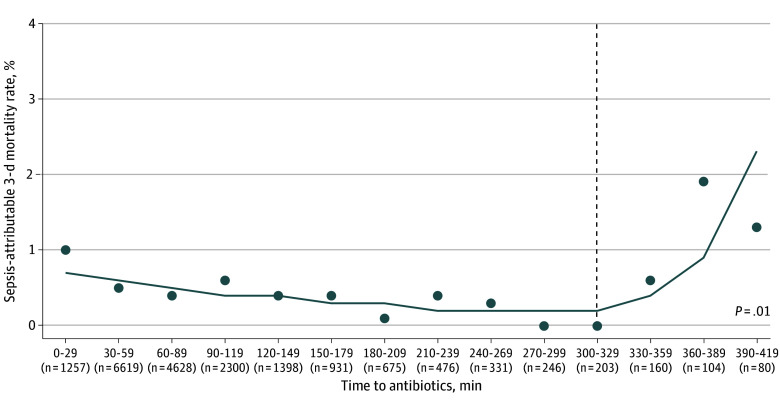
Time to Antibiotics and 3-Day Sepsis-Attributable Mortality Among Children With Sepsis Graph shows piecewise regression analysis evaluating the association of time to antibiotic administration with 3-day sepsis-attributable mortality. Time is represented in 30-minute increments, and the number of patients at each time point is denoted in parentheses. The *P* value represents the change in the unadjusted slope from preinflection to postinflection point (dashed vertical line).

The inflection point in time to antibiotic administration at which 3-day sepsis-attributable mortality changed was 330 minutes from ED arrival (Figure 1). The odds of mortality increased for every 30-minute increment after the inflection point (OR, 2.44; 95% CI, 1.28-4.62; *P* = .01). Overall, the sepsis-attributable mortality was 0.5% (97 patients) at 3 days and 0.9% (170 patients) at 30 days (Table 2). Patients who received an antibiotic in less than 330 minutes (19 164 patients) had sepsis-attributable 3-day mortality of 0.5% (93 patients) and 30-day mortality of 0.9% (163 patients). In unadjusted analyses, the 351 patients who received antibiotics at 330 minutes or later had 3-day sepsis-attributable mortality of 1.2% (4 patients) and 30-day mortality of 2.0% (7 patients). They had more central venous lines placed (46 patients [18.5%] vs 1630 patients [12.9%]) than those who received antibiotics within 330 minutes (Table 2).

**Table 2.** Unadjusted Outcomes of Patients Receiving Antibiotics Before and After the 330-Minute Inflection Point

Outcome	Patients, No. (%)			*P* value^a^
	Total (N = 19 515)	Antibiotic <330 min (n = 19 164)	Antibiotic ≥330 min (n = 351)	
3-d Sepsis-attributable mortality	97 (0.5)	93 (0.5)	4 (1.2)	.08
30-d Sepsis-attributable mortality	170 (0.9)	163 (0.9)	7 (2.0)	.02
ICU admission				
No	10 603 (56.2)	10 410 (56.2)	193 (57.3)	.70
Yes	8255 (43.8)	8111 (43.8)	144 (42.7)	
Not reported	657 (3.4)	643 (3.4)	14 (4.0)	
ICU-free days among ICU-admitted patients, median (IQR)	26 (22-28)	26 (22-28)	26 (22-28)	.10
Ventilator use				
No	12 002 (80.3)	11 783 (80.3)	219 (81.7)	.55
Yes	2948 (19.7)	2899 (19.7)	49 (18.3)	
Not reported	4565 (23.4)	4482 (23.4)	83 (23.6)	
Ventilator-free days among ICU-admitted patients, median (IQR)	26 (20-28)	26 (20-28)	24 (0-28)	.18
Vasoactive medication				
No	16 338 (88.2)	16 053 (88.2)	285 (86.4)	.30
Yes	2189 (11.8)	2144 (11.8)	45 (13.6)	
Not reported	988 (5.1)	967 (5.0)	21 (6.0)	
Vasoactive medication–free days among ICU-admitted patients, median (IQR)	28 (26-29)	28 (26-29)	28 (25-29)	.10
Sepsis days, median (IQR)	5 (3-9)	5 (3-9)	5 (3-8)	.67
Placement of central venous line				
No	11 255 (87.0)	11 052 (87.1)	203 (81.5)	.01
Yes	1676 (13.0)	1630 (12.9)	46 (18.5)	
Not reported	6584 (33.7)	6482 (33.8)	102 (29.1)	

Abbreviation: ICU, intensive care unit.

^a^ Statistical significance was set at 2-sided *P* < .05.

In adjusted analysis, patients who received antibiotics at 330 minutes or later had increased odds of sepsis-attributable mortality at both 3 days (OR, 3.44; 95% CI, 1.20-9.93; *P* = .02) and 30 days (OR, 3.63; 95% CI, 1.59-8.30; *P* = .002) compared with those who received antibiotics before 330 minutes. Other factors associated with increased odds of sepsis-attributable 3-day and 30-day mortality include bacteremia, lactate greater than 36 mg/dL (to convert to millimoles per liter, multiply by 0.111), IPSO critical sepsis, and FTZ based on a huddle or order set (Table 3 and eTable 1 in Supplement 1).

**Table 3.** Multivariable Analysis of 3-Day Sepsis-Attributable Mortality Among Children With Sepsis

Variable	Adjusted probability (95% CI)	Adjusted OR (95% CI)	*P* value
Antibiotic timeliness			
<330 min	0.012 (0.008-0.019)	Reference	NA
≥330 min	0.040 (0.014-0.113)	3.44 (1.20-9.93)	.02
High-risk conditions, technology dependent^a^	0.033 (0.017-0.065)	2.29 (1.41-3.71)	.001
Long-term ventilation^a^			
Yes	0.033 (0.015-0.073)	2.13 (1.18-3.82)	.01
Not reported	0.021 (0.009-0.047)	1.34 (0.62-2.90)	.46
Bacteremia^a^			
Yes	0.042 (0.021-0.082)	2.78 (1.74-4.43)	<.001
Not reported	0.016 (0.006-0.046)	1.06 (0.39-2.86)	.91
Lactate level			
≤36 mg/dL	0.007 (0.003-0.015)	Reference	NA
>36 mg/dL	0.063 (0.032-0.122)	9.38 (5.44-16.17)	<.001
Not reported	0.024 (0.012-0.045)	3.36 (1.92-5.88)	<.001
IPSO sepsis population			
IPSO sepsis	0.010 (0.005-0.020)	Reference	NA
IPSO critical sepsis	0.048 (0.025-0.090)	5.06 (3.10-8.25)	<.001
Functional time zero source			
Screen	0.013 (0.007-0.024)	Reference	NA
Huddle	0.030 (0.012-0.072)	2.42 (1.16-5.05)	.02
Order set	0.029 (0.014-0.056)	2.31 (1.40-3.81)	.001
Arrival time of day			
12:00 am-5:59 am	0.028 (0.013-0.060)	Reference	NA
6:00 am-11:59 am	0.029 (0.015-0.058)	1.06 (0.58-1.96)	.84
12:00 pm-5:59 pm	0.019 (0.009-0.037)	0.66 (0.35-1.25)	.20
6:00 pm-11:59 pm	0.016 (0.007-0.034)	0.56 (0.28-1.10)	.09

Abbreviations: IPSO, Improving Pediatric Sepsis Outcomes; NA, not applicable; OR, odds ratio.

SI conversion factor: To convert lactate to millimoles per liter, multiply by 0.111.

^a^ Answers of no represent referent values.

**Figure 2.**
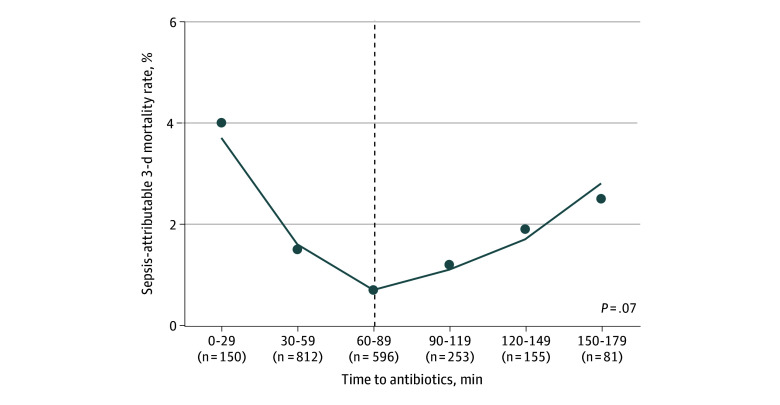
Time to Antibiotics and 3-Day Sepsis-Attributable Mortality Among Children With Bacteremia and Sepsis Graph shows piecewise regression analysis evaluating the association of time to antibiotic administration with 3-day sepsis-attributable mortality in patients with sepsis and bacteremia. Time is represented in 30-minute increments, and the number of patients at each time point is denoted in parentheses. The *P* value represents the change in the unadjusted slope from preinflection to postinflection point (dashed vertical line).

In subgroup analyses, compared with the full cohort, the subgroup with bacteremia (2230 patients [11.4%]) had a higher proportion of patients with IPSO critical sepsis (967 patients [43.4%] vs 6839 patients [35.0%]) and patients with an initial lactate level greater than 36 mg/dL (220 patients [9.9%] vs 1371 patients [7.0%]) (eTable 2 in Supplement 1). Of the 2054 patients with bacteremia who received an antibiotic within 180 minutes of ED arrival (10.5%), the estimated inflection point of time to antibiotic administration at which 3-day sepsis-attributable mortality changed from decreasing to increasing was 90 minutes (Figure 2). The change in slope of mortality rate after this estimated inflection point was not different enough from the preinflection slope to achieve statistical significance. Patients with bacteremia who received antibiotics within 90 minutes had longer lengths of stay and had a higher proportion receiving vasoactive medications, compared with those receiving antibiotics after 90 minutes (eTable 3 in Supplement 1).

In 6839 patients with IPSO critical sepsis (35.0%) and 6725 patients with high-risk conditions (34.5%), the inflection points were similar to those for the main cohort (eFigures 2 and 3 in Supplement 1). eTable 4 in Supplement 1 summarizes mortality rates and relative risk of mortality estimates before and after inflection points for each of the 3 subgroups. In the subgroup analysis of patients who received antibiotics within the first 29 minutes (1265 patients [6.5%]), mortality was higher compared with those who received antibiotics at 30 to 329 minutes (16 patients [1.3%] vs 77 patients [0.4%]) (eTable 5 in Supplement 1).

## Discussion

In this large cohort study of pediatric ED patients treated for sepsis, the sepsis-attributable mortality rate was low (<1%). Overall, sepsis was recognized quickly, and IV antibiotics and fluids were administered promptly. We identified an inflection point in time to antibiotic administration at 330 minutes, beyond which there was an increase in adjusted odds of both 3-day and 30-day sepsis-attributable mortality. ICU admission, vasoactive medication administration, and ventilator use did not differ significantly between patients who received antibiotics before and after the 330-minute inflection point.

These findings support pediatric sepsis guideline recommendations for timely antibiotic administration.^20^ However, the inflection point of 330 minutes at which mortality increased is longer than current recommendations and differs from previously published studies.^11,27^ In a smaller, single-center study of pediatric ICU patients treated for septic shock and/or severe sepsis, Weiss et al^11^ reported an escalating risk of mortality for each hour’s delay in initial antibiotic administration after sepsis recognition, becoming statistically significant after 3 hours (OR, 3.92; 95% CI, 1.27-12.06). Sankar and colleagues^27^ demonstrated higher odds of mortality (OR, 1.83; 95% CI, 1.14-2.92) when antibiotics were administered after 1 hour from sepsis recognition among ED patients with sepsis or septic shock.

One explanation for this difference may be that our study, which focused on ED patients with sepsis recognized within the first hour, may have included patients with sepsis identified earlier in their disease course, when antibiotics could be delayed after arrival but still potentially precede critical deterioration. In addition, because IPSO sepsis definitions are broad, our study likely included a more heterogenous population, including patients who may have been less severely ill and for whom clinical investigation was appropriately prioritized over antibiotic administration. IPSO critical sepsis, which most closely approximates septic shock, was identified in 35% of patients in our study, compared with 79% in the study by Weiss et al^11^ and 77% in the study by Sarkar et al.^27^ These differences in cohort severity of illness likely heavily influenced the time at which mortality became significant as well as the overall mortality rate, with our mortality rate of less than 1% compared with 12%^18^ and 24%.^21^

Given that the initial analysis did not demonstrate an increase in harm until 330 minutes, post hoc analyses were performed investigating important subgroups that might experience harm sooner. In the bacteremic analysis, we identified an inflection point in time of antibiotic administration at 90 minutes. Although this was not a statistically significant finding, patients with bacteremia are most likely to benefit from timely antibiotic administration and should be considered a focus of future investigation. Although there are inherent challenges in prospectively identifying patients with bacteremia early in their clinical course, the method used by Venkatesh and colleagues^28^ to investigate the impact of 1-hour and 3-hour sepsis bundles on outcomes in adult patients with confirmed bacteremia and sepsis could serve as a model for future investigation. In the analyses of patients with IPSO critical sepsis and with high-risk conditions, we identified inflection points similar to that of the main cohort, bolstering the main study findings.

We conducted a post hoc analysis of patients who received antibiotics in the first 29 minutes because of the unexpected finding of higher mortality in this subgroup compared with those who received antibiotics between 30 and 329 minutes. Although unmeasured confounding variables (including severity of illness markers not captured in the database) may have influenced mortality, these patients appear to represent a more critically ill population, and included a higher proportion of patients with malignant neoplasms (eTable 5 in Supplement 1). These patients likely have a higher baseline risk of sepsis mortality, as well as faster time to antibiotics resulting from evidence-based fever and neutropenia protocols requiring antibiotic administration within 60 minutes.^29^ In addition, severely ill children with a very high-risk of mortality at presentation may capture the attention of clinical teams, translating to faster receipt of interventions that may not ultimately improve outcomes given the advanced stage of disease at presentation. Our findings are similar to previously published reports describing increased mortality among pediatric patients receiving antibiotics within the first hour of sepsis recognition.^11,30,31^

Although timely antibiotic administration is important to high-quality sepsis care, sepsis resuscitation is complex and requires substantial resources.^5,20,22,32^ Despite dedicated quality improvement efforts, consistently achieving recommended antibiotic timing thresholds can be challenging.^30,33,34,35,36,37,38^ Prioritizing rapid antibiotic administration at the exclusion of other interventions may not be appropriate for some patients. The Infectious Diseases Society of America task force^39^ opted not to support the 2016 Adult Surviving Sepsis Campaign guidelines that recommend antibiotic administration within 1 hour of sepsis recognition because of concerns that 1 hour may not allow for effective clinical investigation and may result in unnecessary antibiotic administration.^14^ An investigation of a 1-hour sepsis bundle implementation following the New York State mandate found that completion of the entire bundle (obtaining blood cultures, administering broad-spectrum antibiotics, and a 20 mL/kg IV fluid bolus) within 1 hour was associated with lower risk-adjusted odds of in-hospital mortality (OR, 0.59; 95% CI, 0.38-0.93), whereas administration of antibiotics in isolation within 1 hour was not (OR, 0.78; 95% CI, 0.55-1.12).^34^ Similarly, a recently published IPSO interim analysis found that bundle compliant care was associated with significant reduction in mortality.^40^ These results suggest that all bundled-care elements are likely important rather than one singular intervention, which is also emphasized in the 2020 Pediatric Surviving Sepsis Campaign guidelines.^20^

### Limitations

Our study has several limitations. First, this is an analysis of retrospective observational data collected for the purposes of quality improvement. The cohort represents one of largest pediatric sepsis cohorts to date but does not allow for rigorous experimental design. For example, the inclusion of patients according to IPSO criteria who do not have sepsis could have contributed to the later than anticipated inflection point and low mortality rate and may have introduced other confounders. In addition, confounding by indication may underlie the association of time to antibiotic administration with mortality among those receiving antibiotics in less than 30 minutes and 330 minutes or longer. Second, the overall low mortality rate and few patients receiving very late antibiotics may have affected the precision of the inflection point in the piecewise logistic regression analysis. Third, the subgroup of patients with bacteremia, who are most likely to benefit from early antibiotic administration, represented a small subset, limiting robust analysis. Fourth, excluding patients with FTZ greater than 1 hour from ED arrival may limit generalizability to patients with late-onset or hospital-onset sepsis. Fourth, the IPSO database does not capture severity of illness scores, preventing standardized risk stratification, which may limit some conclusions. To mitigate this, we used surrogates, including high-risk conditions and dichotomized initial lactate level, as proxies for severity of illness in our adjusted analysis.

## Conclusions

Among children with sepsis recognized within 1 hour of ED arrival, sepsis-attributable mortality increased with antibiotic administration beyond 330 minutes from ED arrival. The findings are congruent with previous work and guidelines emphasizing that long delays in antibiotic therapy are associated with harm in pediatric sepsis. Future investigation should involve prospectively identifying patients with bacterial blood infections and studying the effect of antibiotic administration time on outcomes.

## Notes

Supplement 1. **eTable 1.** Multivariable analysis of 30-day sepsis attributable mortality among children with sepsis **eTable 2.** Baseline characteristics of children with sepsis and bacteremia **eTable 3.** Unadjusted analysis of outcomes among children with sepsis and bacteremia **eTable 4.** Post hoc subgroup analyses: Inflection points in piecewise regression analysis of the association between time to antibiotics and mortality, and pre- and post-inflection mortality estimates **eTable 5.** Characteristics of patients receiving antibiotics in 0-29 and 30-329 minutes after emergency department arrival **eFigure 1.** Consolidated standards of reporting trials diagram **eFigure 2.** Time to antibiotics and 3-day sepsis attributable mortality among IPSO critical patients **eFigure 3.** Time to antibiotics and 3-day sepsis attributable mortality among patients with high-risk conditions **eAppendix.** Supplemental methods

Supplement 2.
Data Sharing Statement
