# Error in Results

**DOI:** 10.1001/jamanetworkopen.2024.55269

**Published:** 2024-12-16

In the Original Investigation titled “Delays to Antibiotics in the Emergency Department and Risk of Mortality in Children With Sepsis,”^1^ published June 5, 2024, there was an error in the first sentence of the fourth paragraph of the Results section; 52 patients (7.8%) should have been 1371 patients (7.0%). This article has been corrected.^1^

## References

[1] Lane RD, Richardson T, Scott HF, . Delays to antibiotics in the emergency department and risk of mortality in children with sepsis. JAMA Netw Open. 2024;7(6):e2413955. doi:10.1001/jamanetworkopen.2024.13955 38837160 PMC11154154

