# Supplementary material for: Delays to Antibiotics in the Emergency Department and Risk of Mortality in Children With Sepsis
Source: JAMA Netw Open. 2024 Jun 5;7(6):e2413955. doi: 10.1001/jamanetworkopen.2024.13955 (PMC11154154; doi:10.1001/jamanetworkopen.2024.13955)
Supplement: Supplement 1. — eTable 1. Multivariable analysis of 30-day sepsis attributable mortality among children with sepsis eTable 2. Baseline characteristics of children with sepsis and bacteremia eTable 3. Unadjusted analysis of outcomes among children with sepsis and bacteremia eTable 4. Post hoc subgroup analyses: Inflection points in piecewise regression analysis of the association between time to antibiotics and mortality, and pre- and post-inflection mortality estimates eTable 5. Characteristics of patients receiving antibiotics in 0-29 and 30-329 minutes after emergency department arrival eFigure 1. Consolidated standards of reporting trials diagram eFigure 2. Time to antibiotics and 3-day sepsis attributable mortality among IPSO critical patients eFigure 3. Time to antibiotics and 3-day sepsis attributable mortality among patients with high-risk conditions eAppendix. Supplemental methods [file jamanetwopen-e2413955-s001.pdf]

Supplemental Online Content

Lane RD, Richardson T, Scott HF, et al. Delays to antibiotics in the emergency department and risk of mortality in children with sepsis. *JAMA Netw Open*. 2024;7(6):e2413955. doi:10.1001/jamanetworkopen.2024.13955

eTable 1. Multivariable analysis of 30-day sepsis attributable mortality among children with sepsis
eTable 2. Baseline characteristics of children with sepsis and bacteremia
eTable 3. Unadjusted analysis of outcomes among children with sepsis and bacteremia
eTable 4. Post Hoc subgroup analyses: Inflection points in piecewise regression analysis of the association between time to antibiotics and mortality, and pre- and post-inflection mortality estimates
eTable 5. Characteristics of Patients Receiving Antibiotics in 0-29 and 30-329 Minutes After Emergency Department Arrival
eFigure 1. Consolidated Standards of Reporting Trials diagram
eFigure 2. Time to antibiotics and 3-day sepsis attributable mortality among IPSO critical patients
eFigure 3. Time to antibiotics and 3-day sepsis attributable mortality among patients with high-risk conditions
eAppendix. Supplemental Methods

This supplemental material has been provided by the authors to give readers additional information about their work.

© 2024 Lane RD et al. *JAMA Network Open.*

eTable 1. Multivariable analysis of 30-day sepsis attributable mortality among children with sepsis			
	Adjusted Probability (95% CI)	Adjusted OR (95% CI)	p-value
**Antibiotic Timeliness**			
< 330 minutes	0.008 (0.003,0.024)	Reference	
≥ 330 minutes	0.029 (0.008,0.104)	3.63 (1.59,8.30)	0.002
**Age Group**			
29-60 days	0.007 (0.001,0.042)	0.35 (0.08,1.49)	0.16
61 days -364 days	0.027 (0.008,0.085)	1.36 (0.81,2.27)	0.24
1-4 year	0.016 (0.005,0.051)	0.82 (0.55,1.22)	0.32
5-10 years	0.014 (0.004,0.044)	0.71 (0.47,1.07)	0.10
11-17 years	0.020 (0.006,0.060)	Reference	
**High Risk Conditions**[1]			
Not applicable or not reported[1]	0.013 (0.004,0.042	0.70 (0.44,1.11)	0.13
Solid organ transplant[1]	0.008 (0.001,0.058)	0.24 (0.03,1.77)	0.16
Intellectual disability[1]	0.020 (0.006,0.064)	1.67 (1.09,2.56)	0.02
**Chronically Vented**[1]			
Yes	0.021 (0.006,0.069)	1.54 (0.95,2.49)	0.08
Not reported	0.015 (0.004,0.051)	1.15 (0.55,2.40)	0.71
**Bacteremia**[1]			
Yes	0.026 (0.008,0.080)	2.15 (1.47,3.13)	<0.001
Not reported	0.012 (0.003,0.045)	0.99 (0.44,2.24)	0.98
**Lactate**			
Less than or equal to 36 mg/dL[2]	0.008 (0.002,0.025)	Reference	
Greater than 36 mg/dL[2]	0.037 (0.011,0.112)	4.83 (3.26,7.17)	<0.001
Not reported	0.013 (0.004,0.040)	1.63 (1.09,2.45)	0.02
**IPSO Sepsis Population**			
IPSO Sepsis	0.006 (0.002,0.021)	Reference	
IPSO Critical Sepsis	0.037 (0.012,0.111)	6.11 (4.15,8.99)	<0.001
**FTZ Source**			
Screen	0.009 (0.003,0.028)	Reference	
Huddle	0.022 (0.006,0.074)	2.53 (1.39,4.61)	0.003
Order set	0.019 (0.006,0.060)	2.20 (1.46,3.30)	<0.001
**Arrival Time of Day**			
12 am-5:59 am	0.016 (0.005,0.052)	Reference	
6:00 am-11:59 am	0.021 (0.007,0.066)	1.35 (0.81,2.24)	0.25
12 pm-5:59 pm	0.015 (0.005,0.047)	0.95 (0.57,1.58)	0.84
6 pm-11:59 pm	0.011 (0.003,0.037)	0.71 (0.41,1.24)	0.23
**Study Year**			
2017	0.015 (0.004,0.049)	Reference	

© 2024 Lane RD et al. *JAMA Network Open.*

2018	0.021 (0.006,0.066)	1.42 (0.86,2.35)	0.17
2019	0.016 (0.005,0.052)	1.10 (0.66,1.84)	0.72
2020	0.011 (0.003,0.035)	0.72 (0.41,1.29)	0.28
2021	0.016 (0.005,0.053)	1.12 (0.63,1.98)	0.71

CI-confidence interval; OR-odds ratio; IPSO-Improving Pediatric Sepsis Outcomes; FTZ-functional time zero.[1]'No' answers represent referent values. [2]SI unit conversion: To convert lactate to mmol/L, multiply values by 0.111.

© 2024 Lane RD et al. *JAMA Network Open.*

eTable 2. Baseline characteristics of children with sepsis and bacteremia			
	**Total**	**Antibiotic 0-90 minutes**	**Antibiotic > 90 minutes**
N, Episodes	2,230	1,565	665
Time to 1st Abx in minutes, Median (IQR)	65 (46,99)	53 (40,68)	133 (107,184)
Age at FTZ, Median (IQR)	6 (2,12)	6 (2,12)	6 (1,12)
**Age Group, n (%)**			
29-60 days	80 (3.6)	45 (2.9)	35 (5.3)
61 days -364 days	237 (10.6)	148 (9.5)	89 (13.4)
1-4 year	704 (31.6)	516 (33.0)	188 (28.3)
5-10 years	558 (25.0)	396 (25.3)	162 (24.4)
11-17 years	651 (29.2)	460 (29.4)	191 (28.7)
**High Risk Conditions, n (%)**			
Any reported	1,364 (61.2)	1,018 (65.0)	346 (52.0)
Malignancy	472 (21.2)	397 (25.4)	75 (11.3)
Asplenia	52 (2.3)	41 (2.6)	11 (1.7)
BMT	109 (4.9)	92 (5.9)	17 (2.6)
Indwelling line	777 (34.8)	605 (38.7)	172 (25.9)
Solid organ transplant	61 (2.7)	44 (2.8)	17 (2.6)
Intellectual disability	287 (12.9)	188 (12.0)	99 (14.9)
Immunocompromised	580 (26.0)	441 (28.2)	139 (20.9)
Technology dependent	670 (30.0)	478 (30.5)	192 (28.9)
**Chronically Ventilated, n (%)**			
No	1,805 (92.9)	1,255 (93.4)	550 (91.7)
Yes	138 (7.1)	88 (6.6)	50 (8.3)
Not reported	287 (12.9)	222 (14.2)	65 (9.8)
Lactate Value, mg/dL, median[1] (IQR)	18 (9,27)	18 (9,27)	18 (9,27)
**Lactate Category**			
Less than or equal to 36 mg/dL[1]	1,257 (56.4)	912 (58.3)	345 (51.9)
Greater than 36 mg/dL[1]	220 (9.9)	168 (10.7)	52 (7.8)
Unknown/Not reported	753 (33.8)	485 (31.0)	268 (40.3)
Time to 1st hypotension (from arrival), Median (IQR)	95 (16,351)	82 (14,322)	121 (20,428)
Bolus Volume (ml/kg) within 50 minutes, Median (IQR)	18 (0,20)	20 (0,20)	0 (0,19)
Bolus Volume (ml/kg) within 170 minutes, Median (IQR)	40 (30,59)	40 (31,59)	40 (22,51)
Time to 1st Bolus (>5 ml/kg), Median (IQR)	43 (29,66)	38 (27,54)	65 (38,98)
Time to FTZ from arrival, Median (IQR)	13 (8,21)	12 (7,19)	16 (9,27)
**IPSO Sepsis Population, n (%)**			
IPSO Sepsis	1,263 (56.6)	850 (54.3)	413 (62.1)

© 2024 Lane RD et al. *JAMA Network Open.*

IPSO Critical Sepsis	967 (43.4)	715 (45.7)	252 (37.9)
**FTZ Source, n (%)**			
Screen	1,615 (72.4)	1,098 (70.2)	517 (77.7)
Huddle	125 (5.6)	101 (6.5)	24 (3.6)
Order set	490 (22.0)	366 (23.4)	124 (18.6)
**Year, n (%)**			
2017	342 (15.3)	229 (14.6)	113 (17.0)
2018	513 (23.0)	378 (24.2)	135 (20.3)
2019	463 (20.8)	316 (20.2)	147 (22.1)
2020	506 (22.7)	380 (24.3)	126 (18.9)
2021	406 (18.2)	262 (16.7)	144 (21.7)
Arrival Time of Day			
12 am-5:59 am	289 (13.0)	212 (13.5)	77 (11.6)
6:00 am-11:59 am	541 (24.3)	409 (26.1)	132 (19.8)
12 pm- 5:59 pm	759 (34.0)	512 (32.7)	247 (37.1)
6 pm-11:59 pm	641 (28.7)	432 (27.6)	209 (31.4)
**Hospital Type**			
Freestanding children's hospital	1,797 (80.6)	1,273 (81.3)	524 (78.8)
Not Freestanding	433 (19.4)	292 (18.7)	141 (21.2)

[1]SI unit conversion: To convert lactate to mmol/L, multiply values by 0.111. IQR-interquartile range; FTZ-functional time zero; BMT-bone marrow transplant; IPSO-improving pediatric sepsis outcomes

© 2024 Lane RD et al. *JAMA Network Open.*

**eTable 3. Unadjusted analysis of outcomes among children with sepsis and bacteremia**				
	**Total**	**Antibiotic in 0-90 minutes**	**Antibiotic in > 90 minutes**	**p-value[1]**
N, Episodes	2,230	1,565	665	
**3-day sepsis-attributable mortality, n (%)**	30 (1.4)	22 (1.4)	8 (1.2)	0.70
**30-day sepsis-attributable mortality, n (%)**	45 (2.0)	32 (2.1)	13 (2.0)	0.89
**ICU admit, n (%)**				0.45
No	1,101 (49.4)	772 (49.3)	329 (49.5)	
Yes	1,067 (47.8)	745 (47.6)	322 (48.4)	
Not reported	62 (2.8)	48 (3.1)	14 (2.1)	
ICU free days, median (IQR)	26 (20,28)	26 (19,28)	26 (21,28)	0.41
**Ventilator use, n (%)**				0.19
No	1,360 (61.0)	959 (61.3)	401 (60.3)	
Yes	385 (17.3)	280 (17.9)	105 (15.8)	
Not reported	485 (21.7)	326 (20.8)	159 (23.9)	
Ventilator free days among ICU admitted patients, median (IQR)	24 (16,27)	24 (15,27)	25 (18,27)	0.50
**Vasoactive medication, n (%)**				0.04
No	1,709 (76.6)	1,178 (75.3)	531 (79.8)	
Yes	425 (19.1)	312 (19.9)	113 (17.0)	
Not reported	96 (4.3)	75 (4.8)	21 (3.2)	
Vasoactive medication free days among ICU admitted patients, median (IQR)	28 (25,29)	28 (25,29)	28 (25,29)	0.73
**Sepsis days, median (IQR)**	9 (5,15)	9 (5,15)	8 (5,14)	0.01
**Placement of CVL, n (%)**				0.26
No	1,044 (46.8)	722 (46.1)	322 (48.4)	
Yes	284 (12.7)	193 (12.3)	91 (13.7)	
Not reported	902 (40.4)	650 (41.5)	252 (37.9)	

[1]Statistical significance was set at a two-sided p-value of 0.05. ICU-intensive care unit; IQR-interquartile range; CVL-central venous line

© 2024 Lane RD et al. *JAMA Network Open.*

eTable 4. Post Hoc subgroup analyses: Inflection points in piecewise regression analysis of the association between time to antibiotics and mortality, and pre- and post-inflection mortality estimates

	Inflection point	Mortality pre-inflection	Mortality post-inflection	RR Post- vs pre-inflection (95% CI)
Bacteremic (n=2,230)	90 minutes	1.4%	1.2%	0.86 (0.38, 1.91)
IPSO Critical sepsis (n=6,839)	330 minutes	1.1%	2.5%	2.33 (0.58, 9.32)
High risk/chronic ventilator dependence (n=11,476)	330 minutes	0.6%	2.0%	3.71 (1.18, 11.68)

RR- unadjusted relative risk; CI-confidence interval; IPSO-Improving pediatric sepsis outcomes

© 2024 Lane RD et al. *JAMA Network Open.*

eTable 5. Characteristics of Patients Receiving Antibiotics in 0-29 and 30-329 Minutes After Emergency Department Arrival				
	**Total**	**Antibiotic in < 30 minutes**	**Antibiotic in 30-329 minutes**	**p-value**
N, Episodes	19,164	1,265	17,889	
Mortality, n (%)	93 (0.5)	16 (1.3)	77 (0.4)	<0.001
Time to 1st Antibiotic in minutes, median (IQR)	68 (46,111)	25 (21,27)	72 (50,116)	<0.001
Age at FTZ, median (IQR)	6 (2,12)	7 (3,13)	6 (2,12)	0.003
**Age Group, n (%)**				0.04
29-60 days	536 (2.8)	22 (1.7)	514 (2.9)	
61 days -364 days	1,570 (8.2)	87 (6.9)	1,483 (8.3)	
1-4 year	6,066 (31.7)	398 (31.5)	5,668 (31.7)	
5-10 years	4,974 (26.0)	335 (26.5)	4,639 (25.9)	
11-17 years	6,018 (31.4)	423 (33.4)	5,595 (31.3)	
**High Risk Conditions, n (%)**				
Not reported	8,186 (42.7)	596 (47.1)	7,590 (42.4)	0.001
Malignancy	3,638 (19.0)	278 (22.0)	3,360 (18.8)	0.01
Asplenia	612 (3.2)	46 (3.6)	566 (3.2)	0.35
Bone marrow transplant	610 (3.2)	35 (2.8)	575 (3.2)	0.38
Indwelling line	4,021 (21.0)	251 (19.8)	3,770 (21.1)	0.30
Solid organ transplant	611 (3.2)	33 (2.6)	578 (3.2)	0.23
Intellectual disability	3,479 (18.2)	155 (12.3)	3,324 (18.6)	<0.001
Immunocompromised	4,440 (23.2)	288 (22.8)	4,152 (23.2)	0.73
Technology dependent	5,046 (26.3)	269 (21.3)	4,777 (26.7)	<0.001
**Chronically Vented, n (%)**				0.82
No	15,006 (90.5)	836 (90.7)	14,170 (90.4)	
Yes	1,584 (9.5)	86 (9.3)	1,498 (9.6)	
Not reported	2,574 (13.4)	343 (27.1)	2,231 (12.5)	
**Bacteremia, n (%)**				0.39
No	15,591 (87.6)	989 (86.8)	14,602 (87.7)	
Yes	2,200 (12.4)	150 (13.2)	2,050 (12.3)	
Not reported	1,373 (7.2)	126 (10.0)	1,247 (7.0)	
Lactate, mg/dL, median (IQR)[1]	18 (9,27)	18 (9,27)	18 (9,27)	<0.001
Lactate, mg/dL, mean (SE)[1]	21.4 (0.18)	24.6 (0.81)	21.2 (0.18)	<0.001
**Lactate Category, n (%)**				<0.001
Less than or equal to 36 mg/dL (4 mmol/L)	10,970 (57.2)	727 (57.5)	10,243 (57.2)	
Greater than 36 mg/dL (4 mmol/L)	1,353 (7.1)	140 (11.1)	1,213 (6.8)	
Not reported	6,841 (35.7)	398 (31.5)	6,443 (36.0)	
Time to 1st hypotension (minutes from arrival), median (IQR)	68 (14,282)	42 (10,212)	70 (15,288)	<0.001
Bolus Volume (ml/kg) within 50 minutes, median (IQR)	16 (0,20)	20 (19,40)	12 (0,20)	<0.001
Bolus Volume (ml/kg) within 170 minutes, median (IQR)	40 (30,51)	40 (33,55)	40 (30,50)	<0.001

© 2024 Lane RD et al. *JAMA Network Open.*

Time to 1st Bolus (>5 ml/kg), median (IQR)	45 (30,68)	22 (17,28)	47 (33,70)	<0.001
Time to FTZ from arrival, median (IQR)	13 (8,23)	8 (5,14)	14 (8,24)	<0.001
**IPSO Sepsis Population, n (%)**				<0.001
IPSO Sepsis	12,412 (64.8)	748 (59.1)	11,664 (65.2)	
IPSO Critical Sepsis	6,752 (35.2)	517 (40.9)	6,235 (34.8)	
**FTZ Source, n (%)**				<0.001
Screen	14,000 (73.1)	778 (61.5)	13,222 (73.9)	
Huddle	985 (5.1)	61 (4.8)	924 (5.2)	
Order set	4,179 (21.8)	426 (33.7)	3,753 (21.0)	

[1]SI unit conversion: To convert lactate to mmol/L, multiply values by 0.111.IQR-interquartile range; FTZ-functional time zero; SE-standard error; IPSO-improving pediatric sepsis outcomes.

© 2024 Lane RD et al. *JAMA Network Open.*

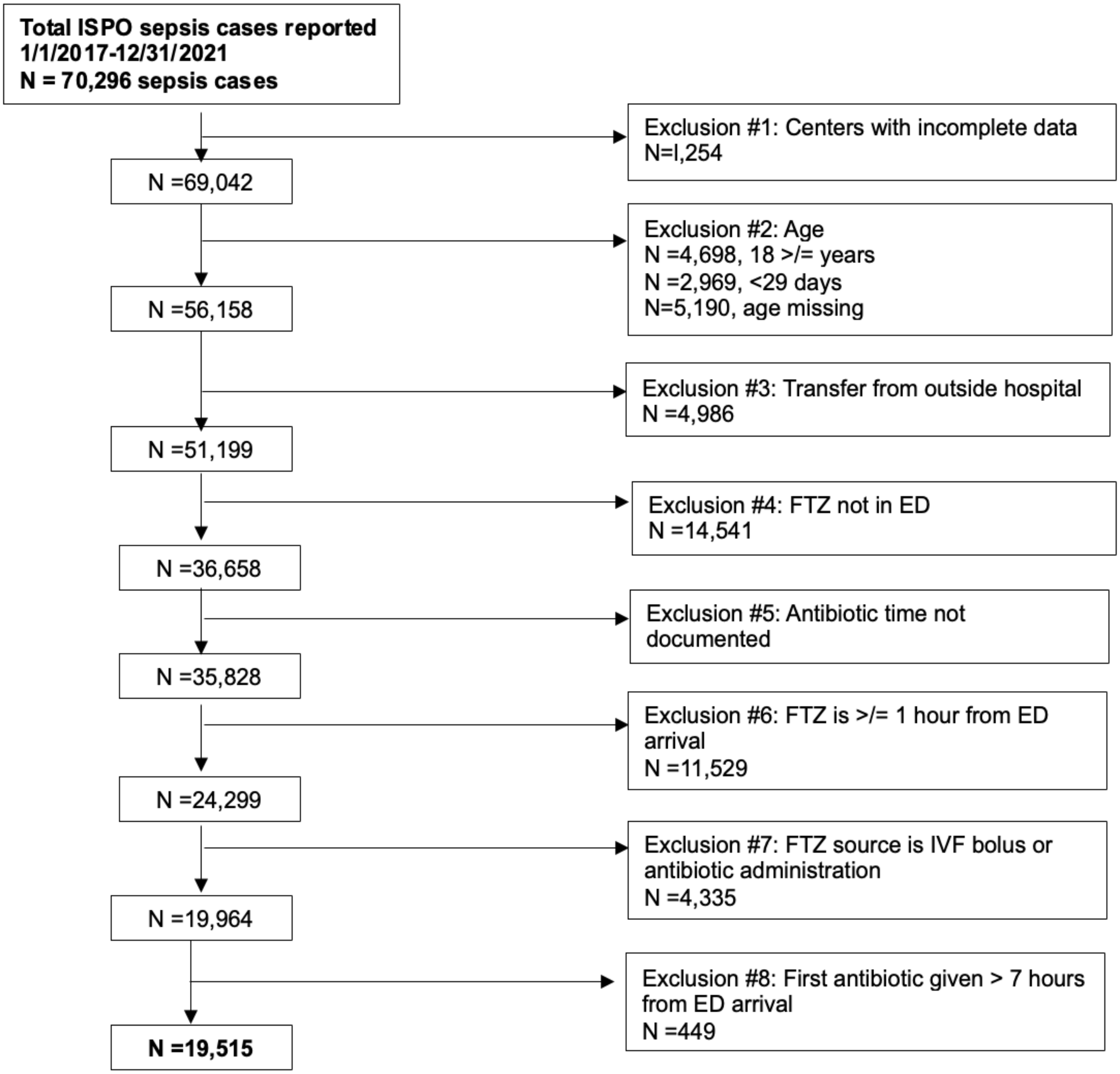

eFigure 1. Consolidated Standards of Reporting Trials diagram

Legend: FTZ-functional time zero; ED-emergency department; IVF-intravenous fluid

© 2024 Lane RD et al. *JAMA Network Open.*

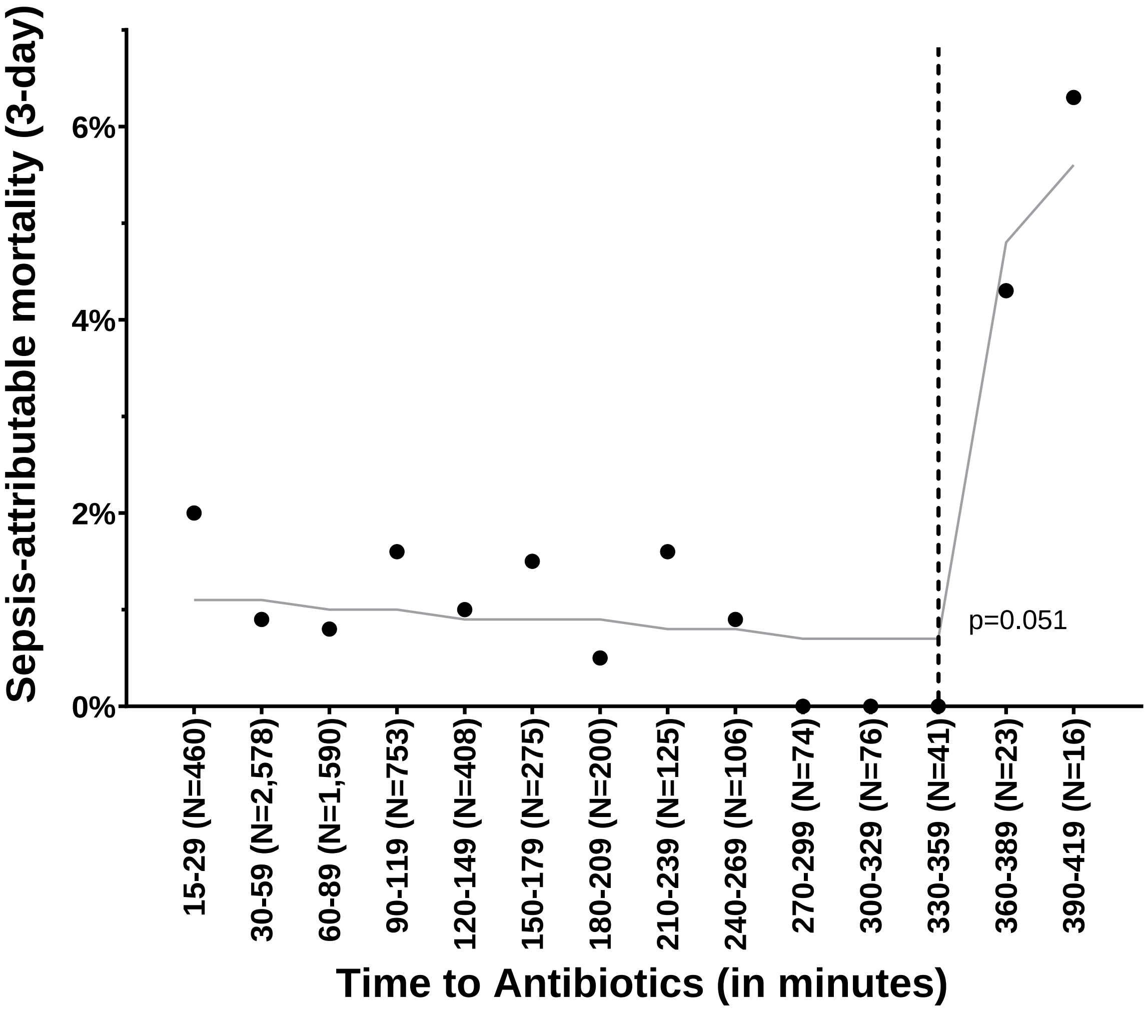

eFigure 2. Time to antibiotics and 3-day sepsis attributable mortality among IPSO critical patients

Legend: Piecewise regression analysis evaluating the association between time to antibiotic administration and 3-day sepsis-attributable mortality in IPSO (Improving Antibiotic Sepsis Outcomes) critical sepsis patients. Time is represented in 30-minute increments and the number of patients at each timepoint is denoted in parentheses. The p-value represents the change in the unadjusted slope from pre- to post-inflection point (dotted vertical line).

© 2024 Lane RD et al. *JAMA Network Open.*

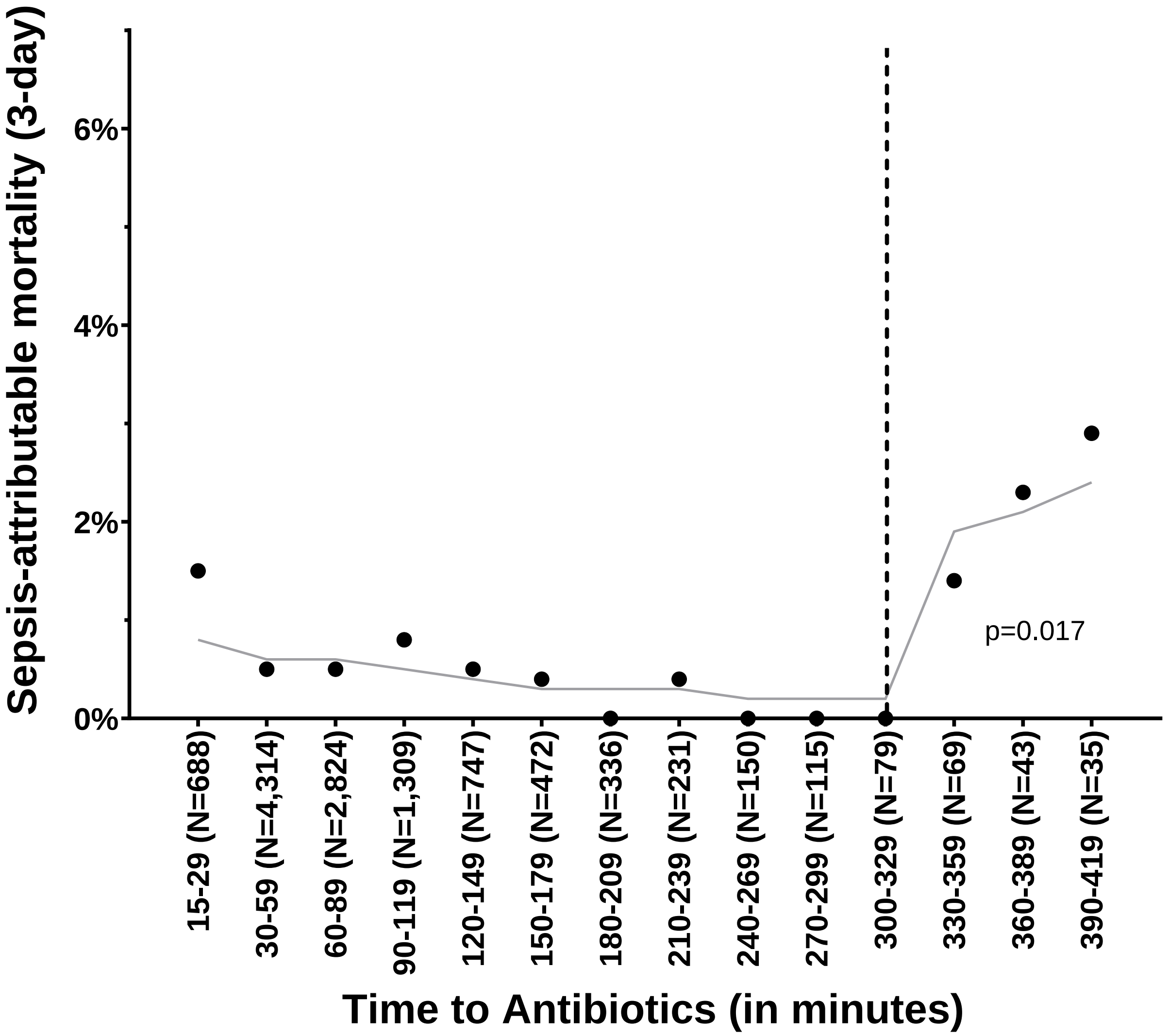

eFigure 3. Time to antibiotics and 3-day sepsis attributable mortality among patients with high-risk conditions

Legend: Piecewise regression analysis evaluating the association between time to antibiotic administration and 3-day sepsis-attributable mortality in patients with high-risk conditions, including chronic ventilator dependence. Time is represented in 30-minute increments and the number of patients at each timepoint is denoted in parentheses. The p-value represents the change in the unadjusted slope from pre- to post-inflection point (dotted vertical line).

© 2024 Lane RD et al. *JAMA Network Open.*

eAppendix. Supplemental Methods

Design/Data Source

Improving Pediatric Sepsis Outcomes (IPSO) sepsis and IPSO critical sepsis cohort case criteria

IPSO definitions are treatment and intervention based and predicated on the collective term, “Treatment” which is defined as, 1) within 6 hours receiving an antibiotic AND either two fluid boluses or one fluid bolus and a vasoactive medication, AND 2) a blood culture within 72 hours.

The IPSO sepsis cohort includes patients who received “Treatment” AND had any one of the following, positive sepsis screen result, sepsis order set used, intensive care unit admission, lactate assessment, vasoactive medication, or other sepsis ICD-10 billing codes. Irrespective of receiving “Treatment”, the IPSO sepsis cohort also includes patients in whom standalone ICD-10 billing codes for severe sepsis with or without septic shock, R65.20 and R65.21, respectively, were used. The IPSO critical sepsis cohort includes a subset of patients meeting IPSO sepsis criteria who received either a third fluid bolus or a vasoactive agent.

Additional details are described in Larsen GY, Brilli R, Macias CG, et al. Development of a Quality Improvement Learning Collaborative to Improve Pediatric Sepsis Outcomes. *Pediatrics*. Jan 2021;147(1) doi:10.1542/peds.2020-1434. https://pubmed.ncbi.nlm.nih.gov/33328337/ and Paul R, Niedner M, Brilli R, et al. Metric Development for the Multicenter Improving Pediatric Sepsis Outcomes (IPSO) Collaborative. *Pediatrics*. May 2021;147(5) doi:10.1542/peds.2020-017889. https://pubmed.ncbi.nlm.nih.gov/33795482/

Improving Pediatric Sepsis Outcomes (IPSO) functional time zero (FTZ)

IPSO describes FTZ as a time-bound metric intended to represent the earliest possible time of sepsis recognition and it is ideally abstracted from the electronic health record (EHR). Because there is no singular biomarker to identify the exact time of sepsis onset, EHR proxies, such as sepsis screen alert time, sepsis huddle time and order set use time, were used. Though not included in this cohort, the IPSO database also identifies time of, first antibiotic administration and first fluid bolus, as surrogates for FTZ. If there is no documentation of a time for sepsis screen, sepsis huddle, order set use, first antibiotic administration, or first fluid bolus, FTZ cannot be assigned.

Additional details are described in, Paul R, Niedner M, Brilli R, et al. Metric Development for the Multicenter Improving Pediatric Sepsis Outcomes (IPSO) Collaborative. *Pediatrics*. May 2021;147(5) doi:10.1542/peds.2020-017889. https://pubmed.ncbi.nlm.nih.gov/33795482/

Exposures and Outcomes

Improving Pediatric Sepsis Outcomes (IPSO) Sepsis-attributable mortality determination and clarification

Sepsis-attributable mortality was assigned to patients who met the IPSO sepsis definition (see above) and whose death was associated with their index sepsis event. The IPSO physician leader(s) from each participating hospital evaluated their mortality cases to determine comorbid conditions and mortality association with a sepsis event. If the patient had comorbid conditions but had not fully recovered from all sepsis related organ dysfunction before death, their death was considered sepsis-attributable. There were 107 (0.5%) patients with non-sepsis-attributable mortality who were excluded from the mortality analyses.

Additional details are described in, Larsen GY, Brilli R, Macias CG, et al. Development of a Quality Improvement Learning Collaborative to Improve Pediatric Sepsis Outcomes. *Pediatrics*. Jan 2021;147(1) doi:10.1542/peds.2020-1434. https://pubmed.ncbi.nlm.nih.gov/33328337/

Per collaborative definitions, patients are followed until discharge or 30-days following functional time zero of sepsis onset. Patients that died within 3-days were also deemed deceased at 30-days. Patients that had not been discharged from the hospital within 30-days of sepsis onset and had not died within 30-days following functional time zero were deemed alive at 30-days for the purposes of the logistic regression model. Hospitals in the collaborative are unable to track patients beyond discharge or readmission.

Statistical Analysis

Multivariable regression model covariate selection details

Initial model covariates (selected *a priori*) included time-to-antibiotic groups, age, high-risk conditions (malignancy, asplenia, bone marrow transplant, indwelling central line, solid organ transplant, severe intellectual disability, immunocompromised status, and technology dependency), chronic ventilator dependence, bacteremia, initial lactate value, IPSO sepsis versus critical sepsis, and FTZ source (screen, huddle or order set).

© 2024 Lane RD et al. *JAMA Network Open.*

Additional variables added after the initial analysis including hospital type, arrival time of day, and year of study. Time to first fluid bolus and hospital type were ultimately excluded due to multicollinearity.

The final 3-day sepsis-attributable mortality model included time-to-antibiotic groups, high risk condition-technology dependency, chronic ventilator dependence, bacteremia, lactate category, IPSO sepsis versus critical sepsis, arrival time of day, and FTZ source (screen, huddle, order set). Several variables fell out during the backward selection process, including study year (p= 0.27).

The final 30-day sepsis-attributable mortality model included time-to-antibiotic groups, age, high risk condition-solid organ transplant and intellectual disability, chronic ventilator dependence, bacteremia, lactate category, IPSO sepsis versus critical sepsis, FTZ source (screen, huddle, order set), arrival time of day, and study year.

Missing variables
If functional time zero or time of first bolus were missing, then these records were excluded from the cohort (eFigure A). In Table 1, we reported the number missing (labeled not reported) variables for high-risk conditions, chronic ventilator dependency, bacteremia, and lactate value.

Improving Pediatric Sepsis Outcomes (IPSO) bacteremia definition
Participating hospitals were asked to review results of blood cultures collected at the index hospital, outside hospitals, and primary care provider clinics and to use their judgement as to whether a positive blood culture was clinically relevant to an IPSO sepsis episode. The timeframe for the positive culture could be from the 48 hours prior to arrival time at the index hospital and up to 72 hours after functional time zero. Participating hospitals then recorded blood cultures as positive or negative in relation to the IPSO sepsis episode in the IPSO database. Hospitals were not required to report the genus and species of the blood culture isolate(s). Since it was beyond the scope of the IPSO collaborative to confirm that positive blood cultures represented true bloodstream infections, we use the term "bacteremia" for patients reported to have positive blood cultures in the IPSO database by the participating hospitals.

Explanation of fluid bolus times of 50 and 170 minutes
In the dataset, only fluid bolus start times are available for analyses; therefore, we limited the analysis to boluses started in the first 50 minutes, allowing roughly 10 minutes to rapidly administer fluid to patients with presumed septic shock. The intent is to approximate fluid actually received by the patient in the first 60 minutes. The same rationale applies to the 170-minute window.

Selection of 30-minute versus 60-minute increment
The 30-minute increment was ultimately selected because we evaluated 60-minute increments versus 30-minute increments to determine the time interval with the most granularity while minimizing the number of increments with 0 mortalities. Therefore, we did not replicate the analysis using 60-minute increments.

© 2024 Lane RD et al. *JAMA Network Open.*
