# Supplementary material for: Delays to Antibiotics in the Emergency Department and Risk of Mortality in Children With Sepsis
Source: JAMA Netw Open. 2024 Jun 5;7(6):e2413955. doi: 10.1001/jamanetworkopen.2024.13955 (PMC11154154; doi:10.1001/jamanetworkopen.2024.13955)
Supplement: Supplement 2. — Data Sharing Statement [file jamanetwopen-e2413955-s002.pdf]

Data Sharing Statement

Lane. Delays to Antibiotics in the Emergency Department and Risk of Mortality in Children with Sepsis. *JAMA Netw Open*. Published June 05, 2024.
doi:10.1001/jamanetworkopen.2024.13955

Data
Data available: No

Additional Information
Explanation for why data not available: We could provide a data dictionary but would be unable to provide individual patient data at this time while the collaborative is still active.
